# The European mesothelioma epidemic

**DOI:** 10.1038/sj.bjc.6690105

**Published:** 1999-02

**Authors:** J Peto, A Decarli, C La Vecchia, F Levi, E Negri

**Affiliations:** Section of Epidemiology, The Institute of Cancer Research, Sutton, Surrey SM2 5NG, UK; Istituto di Statistica Medica e Biometria, Universitá degli Studi di Milano, Milan, 20133, Italy; Istituto per lo Studio e la Cura dei Tumori, Milan, 20133, Italy; Istituto di Ricerche Farmacologiche ‘Mario Negri’, Milan, 20157, Italy; Registre Vaudois des tumeurs, Institut Universitaire de Médecine Sociale et Préventive, CHUV-Falaises 1, Lausanne, CH-1011, Switzerland; London School of Hygiene and Tropical Medicine, London, WC1E 7HT, UK

**Keywords:** mesothelioma, pleural cancer, mortality trends, Europe

## Abstract

Projections for the period 1995–2029 suggest that the number of men dying from mesothelioma in Western Europe each year will almost double over the next 20 years, from 5000 in 1998 to about 9000 around 2018, and then decline, with a total of about a quarter of a million deaths over the next 35 years. The highest risk will be suffered by men born around 1945–50, of whom about 1 in 150 will die of mesothelioma. Asbestos use in Western Europe remained high until 1980, and substantial quantities are still used in several European countries. These projections are based on the fit of a simple age and birth cohort model to male pleural cancer mortality from 1970 to 1989 for six countries (Britain, France, Germany, Italy, The Netherlands and Switzerland) which together account for three-quarters of the population of Western Europe. The model was tested by comparing observed and predicted numbers of deaths for the period 1990–94. The ratio of mesothelioma to recorded pleural cancer mortality has been 1.6:1 in Britain but was assumed to be 1:1 in other countries. © 1999 Cancer Research Campaign


*British Journal of Cancer* (1999) **79**(3/4), 666–672
© 1999 Cancer Research Campaign
Article no. bjoc.1998.0105

### The European mesothelioma epidemic

**J Peto[1,6], A Decarli[2,3], C La Vecchia[2,4], F Levi[5] and E Negri[4]**

[1]Section of Epidemiology, The Institute of Cancer Research, Sutton, Surrey SM2 5NG, UK; [2]Istituto di Statistica Medica e Biometria, Universitá degli Studi di Milano, 20133 Milan, Italy; [3]Istituto per lo Studio e la Cura dei Tumori, 20133 Milan, Italy; [4]Istituto di Ricerche Farmacologiche 'Mario Negri', 20157 Milan, Italy; [5]Registre Vaudois des tumeurs, Institut Universitaire de Médecine Sociale et Préventive, CHUV-Falaises 1, CH-1011 Lausanne, Switzerland; [6]London School of Hygiene and Tropical Medicine, London WC1E 7HT, UK

**Summary** Projections for the period 1995–2029 suggest that the number of men dying from mesothelioma in Western Europe each year will almost double over the next 20 years, from 5000 in 1998 to about 9000 around 2018, and then decline, with a total of about a quarter of a million deaths over the next 35 years. The highest risk will be suffered by men born around 1945–50, of whom about 1 in 150 will die of mesothelioma. Asbestos use in Western Europe remained high until 1980, and substantial quantities are still used in several European countries. These projections are based on the fit of a simple age and birth cohort model to male pleural cancer mortality from 1970 to 1989 for six countries (Britain, France, Germany, Italy, The Netherlands and Switzerland) which together account for three-quarters of the population of Western Europe. The model was tested by comparing observed and predicted numbers of deaths for the period 1990–94. The ratio of mesothelioma to recorded pleural cancer mortality has been 1.6:1 in Britain but was assumed to be 1:1 in other countries.

**Keywords:** mesothelioma; pleural cancer; mortality trends; Europe

The great majority of mesotheliomas are caused by asbestos, and the much higher incidence in men indicates that most are due to occupational rather than environmental exposure. The incidence continues to rise approximately as the third power of time since first exposure to asbestos for many decades after exposure has ceased (Peto et al, 1982), and most patients are men first exposed 30 or more years ago. A country's mesothelioma rate is therefore a quantitative indicator of its population's past exposure – mainly occupational – to asbestos. Mesothelioma is still almost invariably fatal, so analysis of past cohort mortality patterns should provide a reasonably reliable prediction of future trends. A recent analysis of trends in mesothelioma mortality in Britain indicated that the worst-affected cohorts were born in the late 1940s, and that the peak incidence for male mesothelioma will be reached around 2020 (Peto et al, 1995). In contrast, the worst-affected American male generation was born in the late 1920s, the peak will be reached before the year 2000 and mortality will decline rapidly thereafter (Price, 1997). The analysis of trends in Britain was based on the British mesothelioma register, which includes all deaths for which the word 'mesothelioma' appears on the death certificate (OPCS/HSE, 1995). For most countries, the only routinely available national data that correspond reasonably closely to mesothelioma incidence are death rates for cancer of the pleura. We have therefore analysed trends in male pleural cancer mortality for Britain and six other European countries in order to predict European trends in mesothelioma over the next 30 years.

### DATA AND METHODS

Deaths from all causes and from pleural cancer as well as details of resident populations for all European countries were abstracted from the World Health Organization (WHO) database (La Vecchia et al, 1992). Analysis was restricted to countries having a male population of at least 3 million and complete data from 1970 to 1992 or later. After adding data for 1970–71 for The Netherlands and since 1991 for Britain and Italy from national sources, the data were complete up to 1992 for France and The Netherlands, to 1993 for Germany, Italy and Switzerland and to 1994 for Britain. These six countries together account for 72% of the population of Western Europe and accounted for 86% of all male pleural cancer deaths in Western Europe in 1990. Hungary had complete data from 1970 to 1994 and was also included. Two revisions of the International Classification of Diseases (ICD) were used during this period. Pleural cancer was coded 163.0 under the Eighth Revision and 163 under the Ninth Revision.

Age-specific death rates were analysed in 5-year calendar periods beginning in 1970–74 and 5-year age groups from 40–44 to 80–84. We chose the lower age limit of 40 partly because mesothelioma is very rare at younger ages, but also because a disproportionate number of cases in young people may not be caused by asbestos (Peto et al, 1981). Age-standardized rates were based on the world standard population.

### Statistical modelling

The effects of age and cohort of birth were estimated by fitting a log-linear Poisson model to the age-specific death rates for each 5-year calendar period from 1970–74 to 1985–89 using GLIM (Decarli et al, 1987; Peto et al, 1995). Cohorts were defined according to their central year of birth. Thus, for example, individuals dying in the quinquennium 1970–74 aged 80–84, who could have been born in any of the 10 years from 1885 to 1894, were assigned to the 1890 birth cohort. The results of the analysis are summarized for each country by predicted age-specific death rates for men in the 1945 birth cohort, which is the latest cohort for which data prior to 1990 were available, and the cumulative probability (up to age 85) of dying from pleural cancer for each birth

*Received 8 April 1998*
*Revised 16 June 1998*
*Accepted 16 June 1998*

*Correspondence to:* J Peto cohort. Future populations were calculated by standard actuarial methods from the 1985–89 population figures, assuming 1985–89 death rates for all causes for each later period and ignoring migration. Predicted numbers of pleural cancer deaths from 1990 to 2029 were then calculated for each country based on the age-cohort analysis of the data from 1970 to 1989. The validity of the model was tested by comparing observed and predicted numbers of deaths since 1990. Data for this last period were available for 1990–92 for France and The Netherlands, for 1990–93 for Germany, Italy and Switzerland and for 1990–94 for Britain and Hungary. Predicted numbers since 1990 were therefore calculated by interpolation. For example, the central year of birth of French men aged 50–54 in 1990–92 is 1939, so the cohort parameter assumed for this cell was 0.8 of the 1940 plus 0.2 of the 1935 birth cohort parameter estimates. For the purpose of long-term prediction, pleural cancer death rates for the 1950 birth cohort were assumed to equal those of the 1945 birth cohort. The validity of this assumption was assessed by comparing observed and predicted numbers of deaths since 1990 at age 40–44 (Table 2). The rate for the 1955 birth cohort was assumed to be 50% less, and mortality in subsequent birth cohorts was not considered.

Between 1985 and 1989, 74% (2886/3916) of British mesothelioma deaths were classified as pleural (Health and Safety Commission, 1997). (A review of mesothelioma deaths between 1986 and 1991 showed that only 55% were actually coded to ICD 163 as the underlying cause (OPCS/HSE 1995), as mention of another site or cause can take precedence under ICD rules even when pleural malignancy is mentioned. Fifteen per cent were coded as lung, 5% as peritoneal, 21% as other or unspecified cancers and 4% as non-malignant causes). This proportion fell to 62% (3308/5352) in 1990–94 as a result of a change in coding procedure. Systematic medical enquiries to clarify ambiguous causes of death ceased in Britain for deaths registered in 1993 or later (Office for National Statistics, 1996), resulting in a sharp decrease in the number of mesothelioma deaths for which the word 'pleural' was added to the death certificate as a result of such enquiries and a corresponding increase in the number classified as site not specified, which rose from 255 (24%) in 1992 to 603 (49%) in 1994 (Health and Safety Commission, 1997). For the purpose of comparing observed and predicted mortality since 1990, predicted numbers of British pleural cancer deaths in 1990–94 were therefore multiplied by 0.84 (0.62/0.74) to allow for this change (Table 2).

**Table 1** Trends in age-standardized[a] death rates per 100 000 men from pleural cancer in seven European countries, 1970–94

| | Period | | | | | Per cent change |
|---|---|---|---|---|---|---|
| Country | 1970–74 | 1975–79 | 1980–84 | 1985–89 | 1990–94 | in rates |
| Britain | 0.33 | 0.52 | 0.69 | 1.05 | 1.20 | +264 |
| France | 0.68 | 0.87 | 1.08 | 1.36 | 1.42 | +109 |
| Germany | 0.52 | 0.60 | 0.79 | 0.93 | 1.14 | +129 |
| Italy | 0.74 | 0.75 | 0.98 | 1.14 | 1.24 | +68 |
| Netherlands | 0.84 | 1.13 | 1.71 | 2.02 | 2.39 | +184 |
| Switzerland | 0.63 | 0.69 | 0.97 | 1.17 | 1.35 | +114 |
| Hungary | 0.32 | 0.39 | 0.49 | 0.52 | 0.70 | +118 |

[a]World standard population.

### RESULTS

Table 1 shows trends in male age-standardized death certification rates from pleural cancer in the seven countries. In 1970–74 the lowest rates were in Britain and Hungary (0.3 per 100 000) and the highest were in The Netherlands (0.8), France (0.7) and Italy (0.7). Substantial rises were observed in all countries (from +68% in Italy to +264% in Britain). The rate in the period since 1990 was 0.7 in Hungary, 2.4 in The Netherlands and between 1.1 and 1.4 in other countries.

The results of the age-cohort analysis for each country are summarized in Figure 1, which shows predicted age-specific rates for men born in 1945 and the predicted lifetime probability of death from pleural cancer for each birth cohort from 1890 to 1945. Lifetime risks, which are corrected for total mortality, were calculated assuming 1985–89 death rates for all causes for each birth cohort, and within each country are directly proportional to the estimated cohort parameters. The fit of the age-cohort analysis was adequate in each country, the goodness of fit Chi-squared (16 d.f.) ranging from 9.3 ($P = 0.9$) for France to 25.9 ($P = 0.06$) for Italy, with observed and fitted numbers differing significantly (Poisson $P < 0.05$) in only two of the total of 252 age-period cells. The highest risk was suffered by the most recent (1945) birth cohort in most countries. Predicted lifetime probabilities of dying from pleural cancer for men born in 1945 were 0.58% for France, 0.31% for Germany, 0.35% for Italy, 1.39% for The Netherlands, 0.44% for Switzerland, 0.69% for Britain and 0.10% for Hungary.

**Table 2** Observed and predicted male pleural cancer deaths at age 40–44 and at age 45–84 since 1990

| Country | Period | Number of deaths: Age 40–44 Observed | Age 40–44 Predicted | Age 45–84 Observed | Age 45–84 Predicted | Goodness of fit of age + cohort model to data for 1970–89: $\chi^2$ (16 d.f.) | (*P*) |
|---|---|---|---|---|---|---|---|
| Britain | 1990–94 | 46 | 46.5[a] | 2302 | 2407[a] | 14.2 | (0.58) |
| France | 1990–92 | 39 | 45.4 | 1596 | 1724 | 9.3 | (0.90) |
| Germany | 1990–93 | 46 | 48.2 | 2457 | 2324 | 18.7 | (0.28) |
| Italy | 1990–93 | 39 | 46.1 | 2192 | 2079 | 25.9 | (0.06) |
| Netherlands | 1990–92 | 11 | 13.5 | 669 | 695 | 24.4 | (0.08) |
| Switzerland | 1990–93 | 4 | 5.8 | 252 | 250 | 16.4 | (0.43) |
| Hungary | 1990–94 | 14 | 5.4 | 204 | 207 | 14.8 | (0.54) |
| All countries | | 199 | 211 | 9672 | 9686 | | |

[a]Predicted numbers for Britain in 1990–94 have been multiplied by 0.84 to allow for the change in coding procedure described under Data and methods.

© Cancer Research Campaign 1999 

**Table 3** Lifetime pleural cancer risk for men born in 1945–1950 and predicted numbers of pleural cancer deaths in men born in 1955 or earlier

| Country | Lifetime risk of pleural cancer death (%) in men born 1945–50 | Number of deaths per year in 2015–19 | Total number of deaths 1995–2029 |
|---|---|---|---|
| Britain | 0.69 | 1750 | 48 100 |
| France | 0.58 | 1550 | 45 000 |
| Germany | 0.31 | 1370 | 38 900 |
| Italy | 0.35 | 940 | 28 300 |
| Netherlands | 1.39 | 930 | 25 300 |
| Switzerland | 0.44 | 160 | 4 600 |
| Total | | 6700 | 190 200 |

### Mortality in men born in 1950 or later

The only data on men in the 1950 birth cohort are deaths at ages 40–44 occurring since 1990. These observed numbers are compared in Table 2 against those predicted under the assumption that the 1945 and 1950 birth cohort rates are equal. The correspondence is close, both overall (199 deaths, 211 predicted) and for each country except Hungary (14 observed, 5.4 predicted; $P < 0.01$). For the purpose of predicting future numbers of deaths, it was therefore assumed that the 1945 and 1950 birth cohorts will suffer identical rates. The data in Table 2 suggest that this is approximately true both overall and for the six Western European countries. Future numbers of deaths were not calculated for Hungary. The risk to Hungarian men born in 1950 is probably considerably greater than to those born in 1945, but with only 14 deaths in the 1950 birth cohort no reliable projection is possible.

The 1955 birth cohort in each country was assumed to suffer 50% of the 1950 cohort rate, which was the estimate derived from the British mesothelioma register data (Peto et al, 1995). Mortality in subsequent birth cohorts was not considered. This inevitably arbitrary assumption for men born around 1955 and the exclusion of deaths in men born since 1955 have little effect on predicted numbers of deaths up to the year 2020, but subsequent mortality will depend increasingly on the unknown risk to men born since 1955 for whom no data are yet available.

### Observed and predicted mortality since 1990

A projection of future mortality based on past trends may be exaggerated, perhaps severely, by increasing diagnostic awareness of mesothelioma over the last 20 years. We therefore excluded the most recent (1990–94) data from our age-cohort analysis to provide an independent test of our predictions based on the 1970–89 trends. The right-hand part of Table 2 shows observed and predicted numbers of pleural cancer deaths since 1990 in men aged 45–84 for each country. The correspondence is reasonably close (within ± 7%) in each country, and the overall observed and predicted numbers are almost identical (9672 observed, 9686 predicted).

Figure 2 gives the observed numbers of pleural cancer deaths per year in the six Western European countries up to 1989, and predicted numbers up to 2029. For most countries the predicted peak occurs in 2015–19, with about 1750 deaths per year in Britain, 1550 in France, 1370 in Germany, 940 in Italy, 930 in The Netherlands and 160 in Switzerland. These figures are two to three times higher than those in 1990–94. The full impact of the epidemic is summarized in Table 3, which shows for each country the lifetime risk suffered by men born around 1945–50, the number of deaths per year at the peak of the epidemic in 2015–19 and the total number of pleural cancer deaths over the 35 years from 1995 to 2029. The predicted total for the six Western European countries is 190 000 deaths.

### Relationship between pleural cancer and mesothelioma rates

There may be substantial differences between countries in the proportion of deaths from other causes which are incorrectly coded as pleural cancer (ICD 163) and in the proportion of mesothelioma deaths that are coded as pleural cancer. In Britain, 55% (2447/4443) of male mesothelioma deaths in the period 1986–91 were coded 163, and 89% (2447/2745) of death certificates coded 163 included the word mesothelioma and hence were recorded in the mesothelioma register (OPCS/HSE, 1995). Past trends in British pleural cancer rates therefore correspond fairly closely to trends in mesothelioma mortality, the latter being about 162% (0.89/0.55) of the pleural cancer death rate. In France, however, only 70% of a recent sample of male deaths coded to ICD 163 were found on review to be definite, probable or possible mesotheliomas (57% definite or probable, 13% possible). The remaining 30% were attributable to other cancers. In a separate study by the same authors, 75% of male pleural mesothelioma deaths in France were coded 163, suggesting a ratio of pleural mesothelioma to pleural cancer of 0.93 (0.70/0.75) (Brochard et al, 1995). The primary site is peritoneal or undetermined in a substantial proportion of mesotheliomas, however, so the British figure for the proportion of all mesotheliomas that are coded 163 (55%) may be more appropriate. If so, the overall male mesothelioma death rate in France would be about 127% (0.70/0.55) of the pleural cancer mortality rate, implying a current (1998) total of about 1000 male mesothelioma deaths per year in France, and an eventual peak of about 2000.

The observation that 30% of recent male deaths in France coded 163 are not mesotheliomas compared with 11% in Britain may also explain why the overall French pleural cancer rate has exceeded the British rate by a roughly constant amount since 1970 (Table 1). If the number of deaths misclassified as pleural cancer each year has remained roughly constant since 1970, the French age-standardized rates in each period should be reduced by about 20% of the recent French rate, i.e. by about 0.3 per 100 000 per annum, to make them comparable to the British rates. Table 1 shows that this adjustment would make the overall French and British pleural cancer rates almost identical in each period since 1970. This crude

 © Cancer Research Campaign 1999

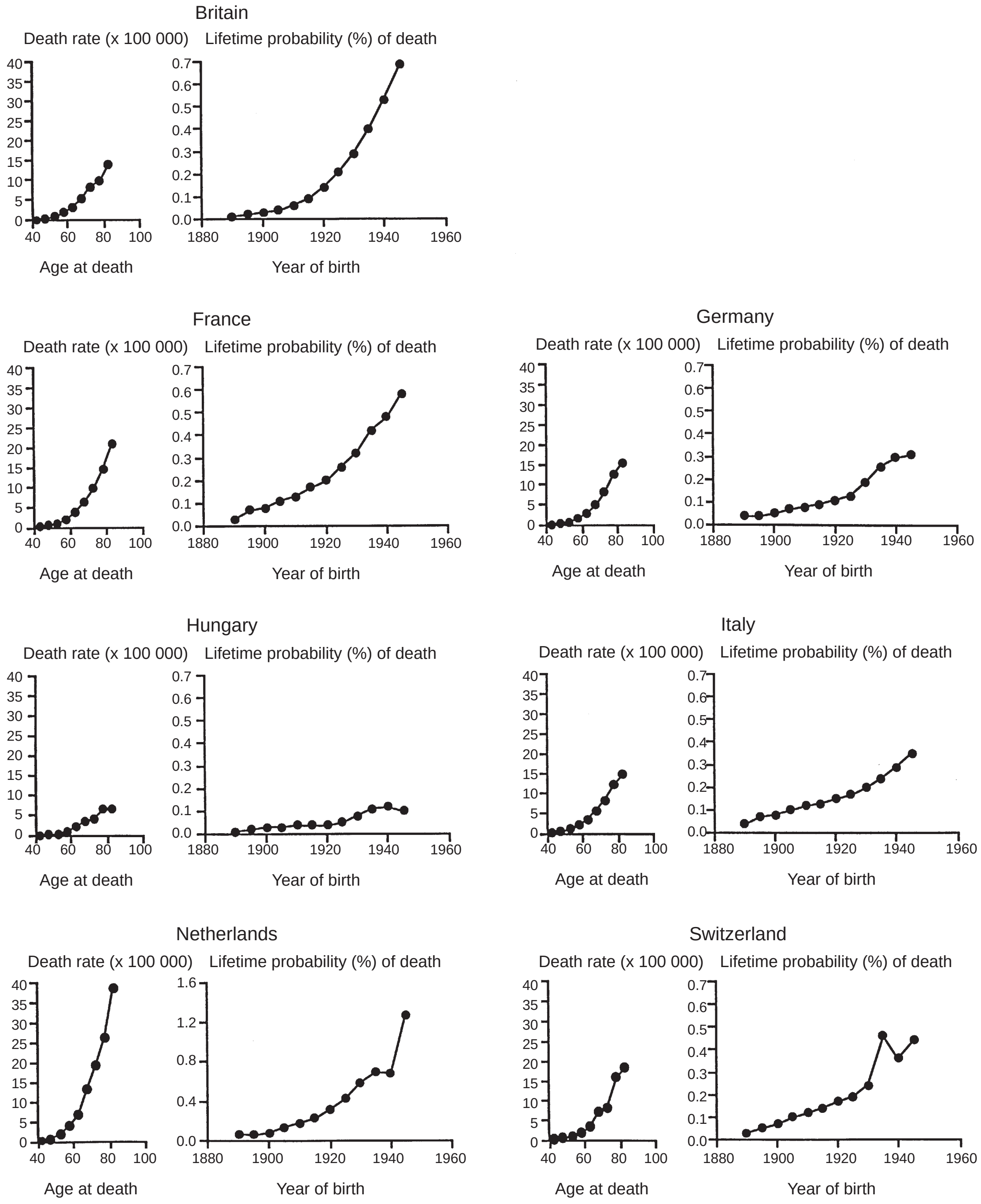

**Figure 1** Results of the age and birth cohort analysis of male pleural cancer mortality in seven European countries. Predicted pleural cancer death rate for men born in 1945 and lifetime probability of dying from pleural cancer (to age 85, allowing for other causes of death) for each birth cohort from 1890 to 1945

correction cannot be applied to age-specific rates, however, as lung cancer, the disease most commonly miscoded as pleural cancer, has exhibited large changes in incidence in Western Europe over recent decades, increasing at older ages and declining at younger ages due to historical changes in cigarette consumption and tar levels (Peto et al, 1994).

There is thus considerable uncertainty in the relationship between recorded pleural cancer and true mesothelioma mortality rates. The apparently marked differences between Britain and France in the overall death rate in 1970–74 and in the rate of increase since 1970 are at least partly due to differences in death certification procedures, but no detailed data on the relationship between pleural cancer and mesothelioma rates are available for other countries. Overall mortality trends in Germany, Italy and Switzerland have been similar to those in France, with age-adjusted rates in the range 0.5–0.7 in 1970–74 increasing to

© Cancer Research Campaign 1999 

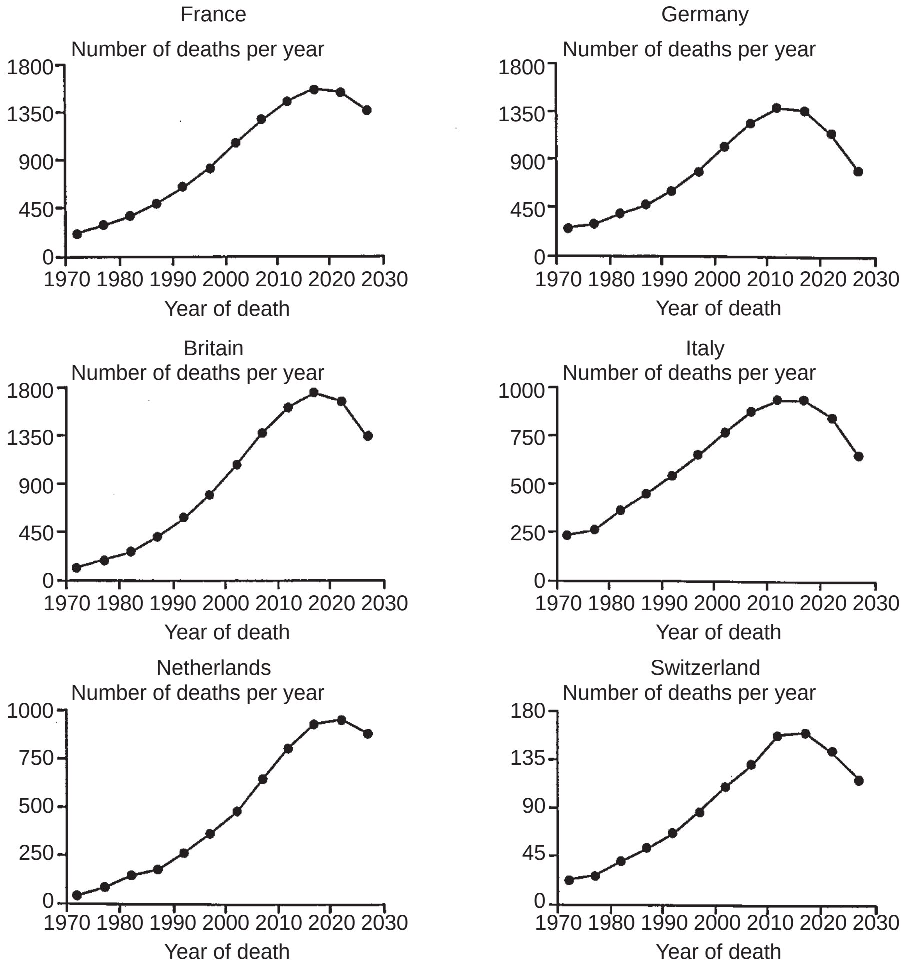

**Figure 2** Observed (to 1989) and predicted (1990–2029) annual numbers of pleural cancer deaths in men in six Western European countries

1.1–1.4 in 1990–94. In the absence of data on the relationship between pleural cancer and mesothelioma mortality comparable to those described above for Britain and France, we have therefore assumed that the ratio of mesothelioma to pleural cancer in the other Western European countries is similar to that in France. For the purpose of predicting mesothelioma trends we have assumed that this ratio is approximately unity, but it could be considerably higher.

### DISCUSSION

The extraordinarily high mesothelioma incidence throughout Western Europe in men born around 1945–50 reflects the extent of asbestos use in the 1960s and 1970s at the beginning of their working lives. Annual raw asbestos imports to European Union countries peaked in the early to mid 1970s and remained above 800 000 tonnes per year until 1980, falling to about 100 000 tonnes by 1993 (European Commission, 1996). Increasingly stringent exposure limits were enforced in the manufacture of asbestos-containing products over this period, but exposure to users of such materials, particularly in the building industry, remained virtually uncontrolled in many countries. Chrysotile asbestos products are still widely used in several European countries, and maintenance or demolition work on older buildings may result in substantial exposure to amphiboles as well as to chrysotile. We have not included men born after 1955 in our projections, but the effects of asbestos exposure during the 1980s and 1990s, although not yet apparent, may prove considerable.

Many European countries introduced legislation to ban the use of crocidolite during the 1970s, and an EC Directive banning the sale and use of all amphiboles has been in force since 1993. An expert committee of the WHO International Programme on Chemical Safety concluded that chrysotile should not be used in construction materials (International Programme on Chemical Safety, 1998). Nine European countries (Denmark, Finland, France, Germany, Italy, The Netherlands, Norway, Sweden and Switzerland) have already banned almost all uses of asbestos of any type; and the British Health and Safety Commission has recently proposed a ban. Most epidemiologists agree that all forms of asbestos can cause both lung cancer and mesothelioma, but there is considerable disagreement on the contribution of chrysotile to overall mesothelioma incidence. In a recent commentary, Cullen (1998) concluded that chrysotile, although less dangerous than amosite and much less so than crocidolite, is probably the main cause of mesothelioma worldwide in view of its far wider use, while McDonald and her colleagues (1997) argued that very few mesotheliomas are caused by pure chrysotile. Decisive evidence remains elusive for several reasons. Few workers have been exposed only to chrysotile, most

© Cancer Research Campaign 1999

historical exposure measurements were of particles rather than fibres, and the interpretation of lung burden studies is complicated by the rapid pulmonary clearance of chrysotile. The controversy has focused on the Quebec chrysotile miners and millers (Liddell et al, 1997; McDonald et al, 1997). The ratio of mesothelioma to excess lung cancer was about 1:4, but the authors attributed most of the 38 mesotheliomas to contamination with tremolite, and concluded that exposure below 300 million particles per cubic foot–years, which was stated to be equivalent to 10 years at 80 fibre $ml^{-1}$, was virtually innocuous. The dose-specific lung cancer risk in this cohort was very much lower than among chrysotile textile workers, who suffered a much higher lung cancer risk than the miners but a low mesothelioma risk (Dement et al, 1983*a*,*b*; McDonald et al, 1983). It is, however, not clear that the enormous particle counts in the Quebec mines constitute a useful measure of exposure to fibres of relevant size (Doll and Peto, 1985). The quantitative effects on risk for each disease of increasing the fibre length from 10 μm to 100 μm or the diameter from 0.02 μm to 0.2 μm are not known, and the large differences in dose-specific lung cancer risk and in the ratio of mesothelioma to excess lung cancer between cohorts exposed mainly to chrysotile may be at least partly due to differences in fibre dimension.

In the absence of a specific ICD code for mesothelioma, we were obliged to base our analyses on the death rate from pleural cancer (ICD 163), an unsatisfactory surrogate that includes a substantial number of lung cancer deaths and excludes mesotheliomas that are not specifically described as pleural on the death certificate. The fit of the age cohort trends up to 1989 and the agreement between observed and predicted numbers since 1990 suggest that ICD 163 has been used fairly consistently in coding deaths over the past 25 years, but there are no satisfactory data on the ratio between pleural cancer and mesothelioma mortality except in Britain. France is the only other country for which any relevant data have been published, and the French surveys were too small to provide precise estimates (178 pleural mesothelioma deaths and a separate survey of approximately 150 deaths coded 163) and did not cover mesotheliomas in which the site of origin was peritoneal or unknown (Brochard et al, 1995). It is thus not clear whether the ratio of mesothelioma to pleural cancer in France and in other European countries is close to unity or considerably higher.

A substantial proportion of recent pleural cancer deaths are not mesotheliomas (11% in Britain and about 30% in France), but this proportion will decline as the number of mesotheliomas increases. The majority of these miscoded deaths are attributable to lung cancer, which is now declining among men (although not among women) in many Western European countries (Peto et al, 1994). Allowance for this would increase our predictions of future mesothelioma mortality, but in the absence of age-specific data on miscoding in each country the appropriate adjustment is not possible. It is unfortunate that the evolution of the epidemic of asbestos-induced mesothelioma, which far exceeds the combined effects of all other known industrial carcinogens, cannot be adequately monitored.

### Future mesothelioma trends in Western Europe

The consistency of the age-cohort pattern in each country suggests that our prediction that the annual number of pleural cancer deaths in Western Europe will more than double between 1990–94 and 2015–19 is reasonably reliable. The prediction for Britain (after adjustment for the ratio between recorded pleural cancer and true mesothelioma rates) is close to that derived from the British mesothelioma register (Peto et al, 1995). Lifetime risks in Britain are particularly low for men born around 1900 and higher than in any country other than The Netherlands for men born since 1940. The pattern in Hungary is atypical, with relatively low risks to men born in 1945 or earlier but evidence of a substantially higher risk to men born in 1950. Cohort trends are generally similar in the remaining four countries, the lifetime risk increasing from about 0.1% for men born in 1905 to between 0.3% and 0.6% for men born in 1945. Data for 1990 in the WHO database show wide variation in other European countries. Recent pleural cancer rates are similar to those presented here in Scandinavia but are about four times less in the remainder of Western Europe.

The predicted number of male pleural cancer deaths for the six Western European countries is 3700 per year for 1998, 6700 per year for 2015–19 and a total of 190 000 between 1995 and 2029 (Table 3). The ratio of male mesothelioma to recorded pleural cancer is about 1.6:1 in Britain and appears to be at least 1:1 in France, and the cohort trends suggest that French death coding practice may be more typical of other European countries. Assuming a ratio of 1:1 for all countries other than Britain would imply a total of 220 000 male mesothelioma deaths between 1995 and 2029 in the six countries. In 1990 these countries accounted for almost three-quarters of the population and accounted for 86% of all male pleural cancer deaths in Western Europe. The number of male mesothelioma deaths in the whole of Western Europe is thus likely to be about 250 000 over the next 30–35 years, and the number of men dying of mesothelioma each year in Western Europe will almost double over the next 20 years, from about 5000 in 1998 to about 9000 in 2018. These estimates suggest that about 1 in 150 of all Western European men born around 1945–50 will die of mesothelioma, and the risk to the occupational groups at highest risk, notably building and engineering workers, must be very much higher.

Our long-term predictions depend crucially on the assumption that the pattern of age-specific rates seen in the past will persist for the next 30 years. In addition to the possible effects of trends in diagnostic completeness, the shape of the age distribution may have been altered by the large reduction in asbestos use that occurred in many countries around 1980. The level of exposure of men born since about 1935 was probably greatly reduced in middle age or earlier, which may eventually cause some flattening of their age-incidence curve compared with that of earlier birth cohorts and thus reduce their eventual risk. The pleural cancer mortality data up to 1994 show no sign of such a change (Table 2), and the numbers of male deaths from 1992 to 1994 in the British mesothelioma register were close both overall and at each age to those predicted by Peto et al (1995) based on the trends up to 1991 (Hodgson et al 1997). The total number of deaths in the register in 1995 was also very close to that predicted, but the 1995 and provisional 1996 data suggest a deficit among men born since 1935 (J T Hodgson, personal communication). Pleural cancer mortality trends in Europe will be disrupted by the introduction of the 10th revision of the ICD, which includes a specific code for mesothelioma, and the most reliable test of our longer term projections, at least for Britain, will be provided by future data from the British mesothelioma register.

The ratio of excess lung cancer to mesothelioma in historical cohort studies of heavily exposed asbestos workers has usually been three or higher (Health Effects Institute, 1991) but evidence from two sources suggests that among less heavily exposed users

© Cancer Research Campaign 1999

of asbestos products the excess of lung cancer may be similar to or even less than the mesothelioma risk. All traceable workers covered by the 1969 UK Asbestos Regulations or the Asbestos (Licensing) Regulations have been followed up through the British Health and Safety Executive's Asbestos Survey (OPCS/HSE, 1995). Among men in the survey there were 729 lung cancer deaths compared with 533 expected. This excess of lung cancer (observed minus expected = 196) was almost equal to the number of mesothelioma deaths (175). British proportional mortality ratios (PMRs) for mesothelioma and lung cancer suggest an even lower ratio of excess lung cancer to mesothelioma in the construction industry. The three building-related occupational groups with the highest PMRs for mesothelioma were plumbers and gas fitters (PMR 443), carpenters (PMR 366) and electricians (PMR 291). The PMRs for lung cancer for these three groups were 107, 94 and 87 respectively (OPCS/HSE, 1995). These surprisingly low ratios may reflect different dose–response relationships related to exposure level or fibre size for these cancers, differences in the long-term pattern of risk many years after exposure has ceased or a disproportionate contribution of amphiboles, particularly crocidolite. Whatever the explanation, it appears that the number of lung cancers caused by asbestos is unlikely to exceed the number of mesotheliomas and may be substantially less.

### ACKNOWLEDGEMENTS

This work was conducted within the framework of the CNR (Italian Research Council) 'Clinical Applications of Oncological Research' (Contracts nos 96.00759.PF39 and 96.00548.PF39) and with the contributions of the Italian Association for Cancer Research, Milan, and the Swiss and Vaud Leagues against Cancer. Professor J Peto was a CNR (A1 97.00133.04) grant recipient. He is supported by the Cancer Research Campaign.

### REFERENCES

Brochard P, Iwatsubo Y, Pairon JC, Pierre N, Boutin C and Bignon J (1995) Estimation of the pleural mesothelioma incidence in France based on the death certificates and the data of a case–control study (abstract). *Third International Mesothelioma Conference*, September 1995 Creteil, France,

Cullen MR (1998) Chrysotile asbestos: enough is enough. *Lancet* **351**: 1377–1378

Decarli A, La Vecchia C, Mezzanotte G and Cislaghi C (1987) Birth cohort, time and age effects in Italian cancer mortality. *Cancer* **59**: 1221–1232

Dement JM, Harris RL Jr, Symons MJ and Shy C (1983*a*) Exposures and mortality among chrysotile asbestos workers. Part I: Exposure estimates. *Am J Ind Med* **4**: 399–419

Dement JM, Harris RL Jr, Symons MJ and Shy C (1983*b*) Exposures and mortality among chrysotile asbestos workers. Part II: Mortality. *Am J Ind Med* **4**: 421–433

Doll R and Peto J (1985) *Asbestos: Effects on Health of Exposure to Asbestos*. Health and Safety Commission: HMSO: London

European Commission and Directorate-General for Industry (1996) *Study on the Impact on the Internal Market of Community Legislation Limiting the Marketing and use of Asbestos*. Environmental Resources Management: London

Health Effects Institute – Asbestos Research (1991) *Asbestos in Public and Commercial Buildings: a Literature Review and Synthesis of Current Knowledge*. Health Effects Institute: Cambridge MA, USA

Health and Safety Commission (1997) *Health and Safety Statistics 1996/97*. HSE Books: Sudbury

Hodgson JT, Peto J, Jones JR and Matthews FE (1997) Mesothelioma mortality in Britain: patterns by birth cohort and occupation. *Ann Occ Hyg* **41** (suppl. 1): 129–133

International Programme on Chemical Safety (1998) Environmental Health Criteria 203: Chrysofile Asbestos. WHO: Geneva

La Vecchia C, Lucchini F, Negri E, Boyle P, Maisonneuve P and Levi F (1992) Trends of cancer mortality in Europe, 1955–1989: II, Respiratory tract, bone, connective and soft tissue sarcomas, and skin. *Eur J Cancer* **28**: 514–599

Liddell FDK, McDonald AD and McDonald JC (1997) The 1891–1920 birth cohort of Quebec chrysotile miners and millers: development from 1904 and mortality to 1992. *Ann Occup Hyg* **41**: 13–36

McDonald AD, Case BW, Churg A, Dufresne A, Gibbs GW, Sebastien P and McDonald JC (1997) Mesothelioma in Quebec chrysotile miners and millers: epidemiology and aetiology. *Ann Occup Hyg* **41**: 707–719

McDonald AD, Fry JS, Woolley AJ and McDonald JC (1983) Dust exposure and mortality in an American chrysotile textile plant. *Br J Ind Med* **40**: 361–367

Office for National Statistics (1996) *Mortality Statistics: Review of the Registrar General on Deaths by Cause, Sex and Age in England and Wales, 1993 (revised) and 1994*. Series DH2 no. 21. HMSO: London

Office of Population Censuses and Surveys/Health and Safety Executive (1995) *Occupational Health Decennial Supplement*. Series DS no. 10. HMSO: London

Peto J, Henderson BE and Pike MC (1981) Trends in mesothelioma incidence in the United States and the forecast epidemic due to asbestos exposure during World War II. In *Quantification of Occupational Cancer*, Banbury Report 9, Peto R and Schneiderman M (eds), pp 51–69. Cold Spring Harbor Laboratory: Cold Spring Harbor, NY

Peto J, Seidman H and Selikoff IJ (1982) Mesothelioma mortality in asbestos workers: implications for models of carcinogenesis and risk assessment. *Br J Cancer* **45**: 124–135

Peto J, Hodgson JT, Matthews FE and Jones JR 1995 Continuing increase in mesothelioma mortality in Britain. *Lancet* **345**: 535–539

Peto R, Lopez AD, Boreham J, Thun M and Heath C (1994) Mortality from smoking in developed countries 1950–2000. Oxford University Press: Oxford

Price B Analyses of current trends in US mesothelioma incidence. *Am J Epidemiol* **145**: 211–218

© Cancer Research Campaign 1999
